# Latent Tuberculosis and Active Tuberculosis Disease Rates among the Homeless, New York, New York, USA, 1992–2006

**DOI:** 10.3201/eid1507.080410

**Published:** 2009-07

**Authors:** John M. McAdam, Scott J. Bucher, Philip W. Brickner, Richard L. Vincent, Steven Lascher

**Affiliations:** St. Vincent’s Hospital, Manhattan, New York, New York, USA (J.M. McAdam, S.J. Bucher, P.W. Brickner, R.L. Vincent, S. Lascher); New York Medical College, Valhalla, New York, USA (J.M. McAdam, P.W. Brickner); Saint Vincent Catholic Medical Center, New York (S. Lascher)

**Keywords:** Tuberculosis and other mycobacteria, latent tuberculosis infection, long-term tuberculosis disease, TB, homeless, New York, USA, dispatch

## Abstract

We conducted a retrospective study to examine trends in latent tuberculosis infection (LTBI) and TB disease rates among homeless persons in shelters in New York, NY, 1992–2006. Although TB case rates fell from 1,502/100,000 population to 0, a 31% LTBI rate in 2006 shows the value of identifying and treating TB in the homeless.

Tuberculosis skin testing (TST) at homeless shelters and drop-in centers is a standard component of tuberculosis (TB) transmission control. Since 1969, St. Vincent’s Hospital has cared for homeless persons in New York City (NYC) (*1*), and 37 shelters and drop-in centers were served in 2006. We report latent tuberculosis infection (LTBI) and TB disease data from the 8 largest of these sites from January 1992 through June 2006. TB screening began at the largest (site 1) in January 1992 and at the others in 1997 through 1999 (Table 1). Because the NYC homeless population is diverse, the populations served by shelters have qualitatively and quantitatively different LTBI risk factors. Sites 1–3 are men’s shelters, site 8 is a women’s drop-in center, and sites 4–7 are drop-in centers for both men and women. At the sites, medical teams assess persons’ TB status using a standard questionnaire and, when indicated, TST. The questionnaire was designed to elicit TB history, prior skin test results, LTBI treatment, and risk factors, including those for progression to TB disease, such as HIV infection. During the time encompassed by this study, St. Vincent’s Hospital initiated 32,108 TB evaluations of homeless persons at these 8 sites; 28,835 (89.8%) were completed.

**Table 1:** Number of tuberculin skin test screenings, by site, New York, NY, USA, January 1992–June 2006

Year	Shelters				Drop-in centers					Total
	1	2	3		4	5	6	7	8	
1992	972	–	–		–	–	–	–	–	972
1993	802	–	–		–	–	–	–	–	802
1994	822	–	–		–	–	–	–	–	822
1995	967	–	–		–	–	–	–	–	967
1996	1,419	–	–		–	–	–	–	–	1,419
1997	1,320	141	45		–	–	–	–	–	1,506
1998	1,208	533	160		2	6	6	–	3	1,918
1999	1,110	550	172		172	167	112	131	151	2,565
2000	1,141	628	114		187	173	166	113	167	2,689
2001	1,153	740	243		180	169	159	91	86	2,821
2002	1,147	717	428		157	141	150	117	85	2,942
2003	1,441	782	441		132	134	96	122	46	3,194
2004	1,548	1,065	209		126	132	86	93	87	3,346
2005	1,958	1,443	78		127	182	145	121	39	4,093
2006*	1,016	722	17		66	91	85	51	4	2,052
Total	18,024	7,321	1,907		1,149	1,195	1,005	839	668	32,108

*January–June.

## The Study

We designed and conducted a retrospective, descriptive study to examine trends in LTBI and TB disease rates among homeless persons at the selected sites. Our dataset was limited to the following categories: age, sex, race/ethnicity, prior TST status, HIV test status, and LTBI treatment history. Those with no previous TST, prior negative tests, or unclear prior test results were offered a TB evaluation. TST was not offered to those with a convincing past history of TB or a documented or convincing history of a previous positive TST result (explanation in the Technical Appendix). We classified each person as follows: TST negative, TST positive as tested, TST positive by history, TB disease, and noncompliant with skin testing reading.

The number of single adult homeless persons identified by the yearly census of the New York City Department of Homeless Services ranged from 27,846 in 1992 to 29,348 in 2005 (*2*), while the number and proportion of TST screenings by our program increased from 972 (3% of New York City total) in 1992 to 4,093 (13.9%) in 2005, the last complete year of our data.

**Figure 1:**
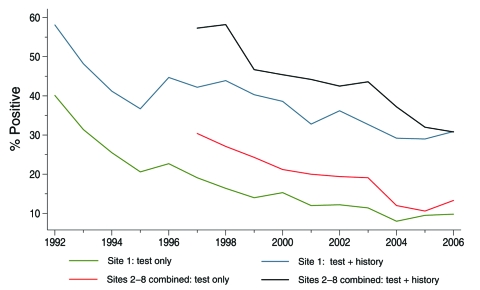
Tuberculin skin test positivity by site and year, New York, NY, USA, January 1992–June 2006.

Among the 32,108 attempted TB screenings, 28,835 persons (89.8%) completed the screening process; 11,385 had positive TST results or history (Table 2). We stratified TST data from all sites and compared those from site 1 (58.1% of observations) to those from sites 2–8 combined, for all years. Using a nonparametric test for trend across ordered groups (developed by Cuzick [*3*] as an extension of the Wilcoxon rank-sum test), we found the 4 testing groups each had statistically significant negative trends by year: site 1 test only, z = –3.64, p<0.001; site 1 test and history, z = –3.35, p = 0.001; sites 2–8 test only, z = –2.91, p = 0.004; sites 2–8 test and history, z = –2.95, p = 0.003 (Stata 10.1 manual: nptrend test) (*4*). The statistically significant decreasing trends for the TST and TST positive by history groups for both site 1 and the other sites combined, from the inception of the data collection, are shown in Figure 1.

**Table 2:** Results of TB screenings, New York, NY, USA, January 1992–June 2006*

TB screening status	No. (%)
Noncompliant with skin test reading	3,273 (NA)†
TST–	17,368 (60.4)
TST+	3,597 (12.5)
TST+ by history	7,788 (27.1)
Active TB	63 (<1.0)
Anergic‡	19 (<1.0)
Total completing evaluation	28,835 (100.0)

*TB, tuberculosis; TST, tuberculin skin testing; NA, not applicable. †The denominator includes all those persons for whom TST status became known. Those noncompliant with TST interpretation have not been included. Thus, in the Technical Appendix Table 4, the percentage of such persons is NA. ‡No reaction to TST, mumps virus, *Candida* spp.

Overall, when the categories of administered TST and TST positivity by history were combined as indicators of LTBI, results decreased from 58.1% to 30.9% (1992–2006) at site 1 and from 57.3% to 30.8% at sites 2–8 combined (1997–2006) (Technical Appendix Table 1). Since few persons with a history of TST provided documentation, we examined LTBI rates among those persons actually tested. By this criterion, TST positivity decreased from 40.1% to 9.8% at site 1 (1992–2006) and from 30.4% to 13.3% at sites 2–8 combined (1997–2006).

**Figure 2:**
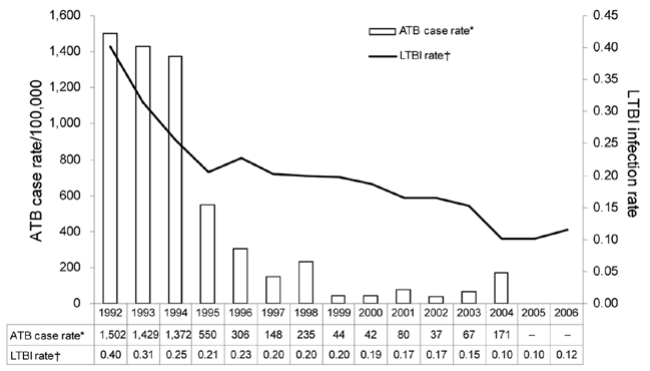
Tuberculosis disease case rates (per 100,000) and infection rates (as tested) by year, New York, NY, USA, 1992–2006. ATB, active tuberculosis; LTBI, latent tuberculosis infection.

TB disease rates at all 8 sites combined (Figure 2) also decreased in the screened population over the study period. Sixty-three persons with TB disease were identified from January 1992 through June 2006. In 1992, the calculated case rate was 1,502/100,000 population compared with 171/100,000 in 2004. These rates are far greater than for the general New York City population (13.0/100,000) and the United States population (4.4/100,000) in 2007 (*5*). From January 2005 through June 2006, no cases were identified. Treatment history was included in the TB questionnaire starting in 1996. The percentage of persons with a history of positive TST and who received >6 months of treatment trended upward, from 50.0% in 1996 through 1997 to 84.2% in 2006 (Technical Appendix Figure).

Using logistic regression, we modeled the risk factors (Technical Appendix Tables 1–4), and the probability of being TB positive (by test or history) and of not having the TB test read. For TB positivity, the adjusted odds ratio (OR) for each age group compared with that for the youngest age group was significant and increased linearly with age. In general, the OR increased by a factor of 2 for each advancing age group (Technical Appendix Table 3). Regarding compliance in TST reading, being in an older group was protective (Technical Appendix Table 3).

We were able to assess changes in HIV-positivity rates from January 2001 through June 2006. The percentage fluctuated between 2.1% and 3.1%. The stage of HIV disease (from asymptomatic HIV-antibody positive to AIDS) was not recorded. For comparison, during this period in New York City, the number of persons who received a diagnosis of AIDS each year decreased from 5,616 to 3,672 (*6*). Because the percentage of HIV antibody positivity among those screened was essentially constant during the study period, we believe that the decreasing LTBI rates (represented by TST positive as tested) are real and not due to increasing numbers of persons with advanced HIV disease who are unable to mount an appropriate skin test response.

Our acceptance of a compelling history of prior positive TST rather than retesting may have resulted in inaccurate reporting of LTBI rates. However, we decided to accept compelling histories on the basis of decades of clinical experience in caring for homeless persons, experience in completing forms in the same way over the study years, and by a desire to do no harm. Our population is not a random sample and may not represent the total homeless population within the city, raising concern about the generalizability of our findings. Also, because the results of all TST screenings were included in our analysis, persons who were screened multiple times are overrepresented. Of 32,108 attempted screenings, 4,724 (14.7%) persons received >1 evaluation. Also, the number of screenings at each site over the years varied, depending on changing requirements of the shelter program.

In the past 2 decades, major public health efforts have been made to evaluate and treat persons with both TB disease and LTBI. Our analysis offers evidence that these attempts to control TB in the homeless population have been beneficial (*7*–*9*).

Although case rates of TB disease are carefully measured by public health authorities throughout the United States, this is not true for LTBI rates. Therefore, we cannot directly compare the rates of LTBI among these homeless persons to those among the general population. However, the rates of LTBI and TB disease observed in this study may serve as a valuable resource for TB control planning.
